# Long Non-coding RNAs in Endothelial Biology

**DOI:** 10.3389/fphys.2018.00522

**Published:** 2018-05-14

**Authors:** Tyler Weirick, Giuseppe Militello, Shizuka Uchida

**Affiliations:** Cardiovascular Innovation Institute, University of Louisville, Louisville, KY, United States

**Keywords:** bioinformatics, databases, lncRNAs, miRNAs, RNA-seq, RNA editing, RNA modifications

## Abstract

In recent years, the role of RNA has expanded to the extent that protein-coding RNAs are now the minority with a variety of non-coding RNAs (ncRNAs) now comprising the majority of RNAs in higher organisms. A major contributor to this shift in understanding is RNA sequencing (RNA-seq), which allows a largely unconstrained method for monitoring the status of RNA from whole organisms down to a single cell. This observational power presents both challenges and new opportunities, which require specialized bioinformatics tools to extract knowledge from the data and the ability to reuse data for multiple studies. In this review, we summarize the current status of long non-coding RNA (lncRNA) research in endothelial biology. Then, we will cover computational methods for identifying, annotating, and characterizing lncRNAs in the heart, especially endothelial cells.

## Introduction

**Figure 1:**
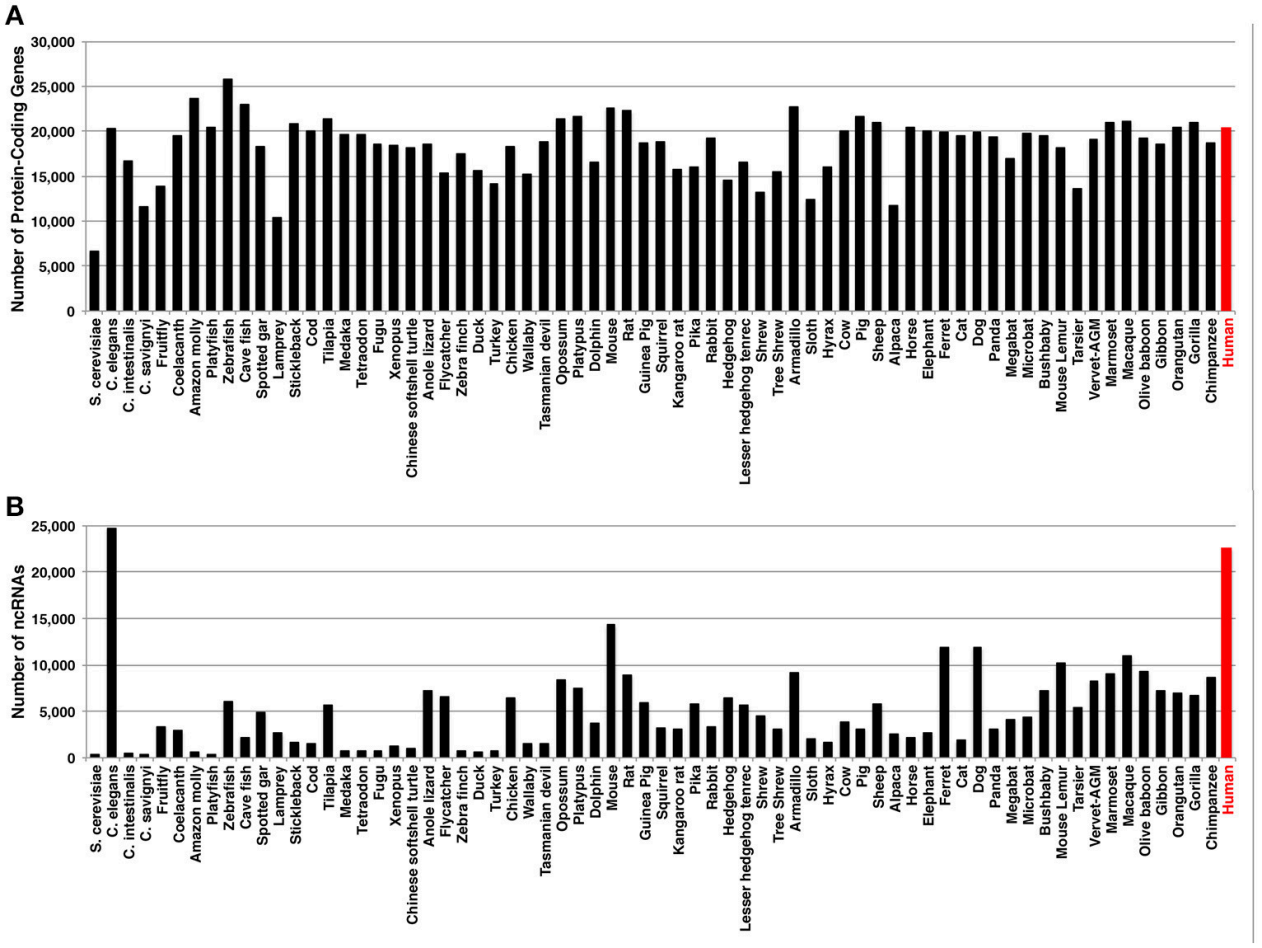
Numbers of **(A)** protein-coding genes and **(B)** ncRNAs, including miRNAs and lncRNAs. The information is based on the Ensembl database (Accessed on May 5, 2017).

The development of next generation sequencing (NGS) and RNA sequencing (RNA-seq) has significantly improved the understanding of transcriptomes. For example, we now know that most of the human genome is transcribed (Lander et al., 2001), yet only a small percent of these RNAs code for protein (Weirick et al., 2016a). When the human genome was annotated (i.e., giving the definition to the genome by naming a particular gene and its corresponding exons), it was originally thought that the number of protein-coding genes in humans should be more than those of lower organisms (e.g., yeast, plants, fishes, amphibians) (Mercer et al., 2011; Ezkurdia et al., 2014). However, when the numbers of protein-coding genes are compared among species, the number of human genes is not more than those of lower organisms (Figure 1A). Given that humans are able to carry out more complex tasks than lower organisms, the question remains in the field: What aspect of our genome allows for the increased complexity? One school of thoughts is that proteins can be modified for various biological processes (e.g., phosphorylation of a protein for its activation). Another school suggests for the increased variety of isoforms resulting from one gene due to the alternative splicing events. In both schools, the ultimate final products are proteins as we know more about proteins than RNAs. Last school postulates that the increased number of ncRNAs (especially, lncRNAs) is at the base of the highest complexity in human, although it is highly subjective as the number of ncRNAs depends on how well the organism is studied as *C. elegans* has more lncRNAs than any other organisms (Figure 1B). At the moment, it is most likely that the combination of these schools of thoughts may yield important answers to the question.

In recent years, many review articles about lncRNAs in the heart are published (Geisler and Coller, 2013; Archer et al., 2015; Devaux et al., 2015; Iyer et al., 2015; Ounzain et al., 2015a; Philippen et al., 2015; Rizki and Boyer, 2015; Uchida and Dimmeler, 2015; Ballantyne et al., 2016; Busch et al., 2016; Lorenzen and Thum, 2016; Uchida and Bolli, 2017; Viereck and Thum, 2017; Sallam et al., 2018). Furthermore, there are large amounts of screening data available for lncRNAs expressed in the heart (Ounzain et al., 2014, 2015b,c; Kurian et al., 2015) even single-cell RNA-seq data (Chan et al., 2016; Delaughter et al., 2016; King et al., 2017; Lescroart et al., 2018; Skelly et al., 2018). However, these data sets were mostly generated for specific purposes and have largely not been analyzed for lncRNAs. Here, we will focus on endothelial cells (ECs), an important cell type in cardiovascular medicine. Furthermore, we will cover computational methods for identifying, annotating, and characterizing lncRNAs in the heart, especially ECs.

## lncRNAs in endothelial cells

Vessels deliver metabolites and oxygen to the tissue and export waste products to sustain the well-being of an organism (Asahara et al., 2011). After tissue injury [e.g., myocardial infarction (MI)], ECs migrate to the site of injury to re-establish the capillary network through a process called “angiogenesis” (Jakobsson et al., 2010; Oka et al., 2014). Furthermore, ECs contribute to the multicellular communications that maintain the balance between the regeneration and dysfunctional or maladaptive healing (Cines et al., 1998; Libby, 2012; Kluge et al., 2013; Eelen et al., 2018). Although advances have been made to understand angiogenesis, the recent emergence of lncRNAs has added another layer of complexity to the genetic network of angiogenesis. To date, a number of lncRNAs are identified and characterized (Table 1) as ECs can be found throughout the human body. For example, *MALAT1* regulates endothelial cell function and vessel growth via cell cycle control (Michalik et al., 2014); the histone demethylase JARID1B controls the lncRNA *MANTIS*, which regulates EC function and vessel growth by binding to the chromatin modifying enzyme BRG1 (Leisegang et al., 2017); and several lncRNAs bind miRNAs to function as miRNA sponges (He et al., 2015, 2017; Huang et al., 2015; Yan et al., 2015; Lu et al., 2016; Ming et al., 2016; Ma Y. et al., 2017; Sun et al., 2017; Zhang B. Y. et al., 2017; Bao et al., 2018).

**Table 1:** List of lncRNAs in endothelial cells.

**LncRNA**	**Organism**	**Tissue**	**Cell type**	**Function**	**References**
*ALT1*	Human	N/A	HUVEC	Interacts with ACE2 and CUL1 to control the expression of Cyclin D1 possibly via ubiquitinatiion and degradation.	Li et al., 2017b
*ASncmtRNA-2*	Human; mouse	Aortas of old mice	HUVEC	Might be involved in the RS establishment by participating in the cell cycle arrest in G2/M phase, possibly through the production of *miR-4485* and -*1973*.	Bianchessi et al., 2015
*GAS5*	Human	Atherosclerotic plaques	HUVEC	Can be transferred from macrophages to EC in exosomes to induce apoptosis of ECs.	Chen et al., 2017
*GATA6-AS*	Human; mouse	Cell-based Xenograft model	HUVEC	Binds the lysyl oxidase LOXL2 to impair its function as H3K4me3 deaminase.	Neumann et al., 2018
*H19*	Human	Brain; Glioma tissue specimens	HBMVEC	Knockdown of *H19* suppressed glioma induced angiogenesis by inhibiting *miR-29a*, which may modulate the onset of glioma by regulating biological behaviors of glioma vascular ECs.	Jia et al., 2016
*H19*	Human	N/A	HUVEC	Is contained in exosomes released by CD90+ cancer cells to promote angiogenic phenotype and cell-to-cell adhesion in ECs.	Conigliaro et al., 2015
*HIF1A-AS2*	Human; rat	Permanent middle cerebral artery occlusion model	HUVEC	Facilitates the up-regulation of *HIF-1α* by sponging *miR-153-3p*, thereby promoting angiogenesis in hypoxia.	Li et al., 2017a
*HOTAIR*	Human	N/A	HBMVEC	Is contained in the glioma cell-derived extracellular vesicles and transmitted into ECs.	Ma X. et al., 2017
*HOTAIR*	Human	Atherosclerotic plaques	HUVEC; HAEC	Positively regulates proliferation and migration of ECs.	Peng et al., 2017
*HOTTIP*	Human	CAD and normal arterial tissues	HUVEC	Its overexpression induces β-catenin expression and enhances the downstream protein c-Myc expression in ECs to affect cell proliferation and migration.	Liao et al., 2018
*IGF2AS*	Rat	Heart	mMVE	Reciprocal regulation *IGF2AS* and *IGF2* is critical in modulating angiogenic development in myocardial tissues in type 2 diabetes.	Zhao et al., 2017
*LEENE*	Human; mouse	Thoracic aorta and aortic arch	HUVECs; HAoEC	Serves as a guide to facilitate RNA Pol II binding to the promoter of *eNOS*.	Miao et al., 2018
*LINC00305*	Human	N/A	HUVEC	Binds *miR-136* to control apoptosis.	Zhang B. Y. et al., 2017
*LINC00341*	Human	N/A	HUVEC	Guides EZH2 [the catalytic subunit of polycomb repressive complex 2 (PRC2)] to the promoter region of the *VCAM1* gene to suppress VCAM1.	Huang et al., 2017
*LINC00657*	Human	N/A	HUVEC	Binds *miR-590-3p* to attenuate the suppression of *miR-590-3p* on *HIF-1a*, and to promote angiogenesis through VEGF, MMP-2, and MMP-9.	Bao et al., 2018
*lincRNA-p21*	Mouse	N/A	mouse lymphoid endothelial cell line SVEC4	Binds *miR-130b* to promote cell apoptosis and induce cell cycle progression.	He et al., 2015
*LISPR1*	Human	N/A	HUVEC; HAoEC; HMEC	Acts as a novel regulatory unit important for S1PR1 expression and EC function.	Josipovic et al., 2018
*LOC100129973*	Human	N/A	HUVEC	Binds *miR-4707-5p* and -*4767*, which promote apoptosis by targeting and downregulating two apoptosis inhibitors, API5 and BCL2L12, respectively.	Lu et al., 2016
*MALAT1*	Human	Peripheral blood from patients diagnosed with unstable angina	HUVEC	Protects the endothelium from ox-LDL-induced endothelial dysfunction partly through competing with *miR-22-3p* for endogenous RNA.	Tang et al., 2015
*MALAT1*	Human; mouse	Mouse retinal angiogenesis model	HUVEC	Regulates EC function and vessel growth via cell cycle control.	Michalik et al., 2014
*MALAT1*	Human	N/A	HUVEC	Binds *miR-320a*, which targets the pro-proliferative gene *FOXM1* for ECs.	Sun et al., 2017
*MALAT1*	Rat	Retina of diabetic rats	Monkey choroid, retina cell line RF/6A	Regulates EC function via p38 MAPK signaling pathway.	Liu et al., 2014
*MALAT1*	Human; mouse	Kidneys of diabetic mice	HUVEC	Regulates glucose-induced up-regulation of inflammatory mediators IL-6 and TNF-α through activation of SAA3.	Puthanveetil et al., 2015
*MANTIS*	Human	Brain microvessel isolation from glioblastoma patients	HUVEC; HAoEC; HDLEC; PAEC	Regulates EC function and vessel growth by binding to the chromatin modifying enzyme BRG1.	Leisegang et al., 2017
*Meg3*	Mouse; monkey	Retina of diabetic mice	Monkey choroid, retina cell line RF/6A	Activates PI3k/Akt signaling.	Qiu et al., 2016
*Meg3*	Rat	Brain	RBMVEC	Physically interacts with p53, which binds to the promoter of *Nox4* to regulate cell growth and the blood vessel growth factor expression.	Zhan et al., 2017
*MEG3*	Human	N/A	HUVEC	Is regulated by HIF-1α to maintain VEGFR2 expression in ECs and plays a vital role for VEGFA-mediated endothelial angiogenesis.	Ruan et al., 2018
*MEG3*	Human; mouse	Hind-limb ischemia in aged mice	HUVEC	Its silencing prevents aging-mediated inhibition of sprouting activity.	Boon et al., 2016
*MEG3*	Human; mouse	Circulating ECs from metabolic syndrome (MetS) patients	EPC	Protects ECs via decreasing *miR-140-5p* expression and increasing HDAC7 expression in MetS.	Liu H. Z. et al., 2016
*MEG3*	Human	N/A	HUVEC	Binds *miR-9* to control the proliferation and angiogenesis of ECs.	He et al., 2017
*MIAT*	Human; rat	Diabetes mellitus	HUVEC; HMVEC	Binds *miR-150-5p* to regulate EC function by forming a feedback loop with VEGF.	Yan et al., 2015
*PUNISHER*	Human; zebrafish	Heart	HUVEC	Its inhibition results in severe vascular defects in zebrafish embryos and reduced cell proliferation in HUVEC.	Kurian et al., 2015
*PVT1*	Human	N/A	Human cerebral microvascular endothelial cell line hCMEC/D3	Binds *miR-186*, which targets *Atg7* and *Beclin1* mRNAs.	Ma Y. et al., 2017
*RNCR3/LINC00599*	Human; mouse	Aortic atherosclerotic lesions	HUVEC	Forms a feedback loop with KLF2 and *miR-185-5p* to regulate EC function.	Shan et al., 2016
*SENCR*	Human	N/A	HUVEC	Induces proliferation, migration, and angiogenesis.	Boulberdaa et al., 2016
*SIRT1 AS lncRNA*	Mouse	N/A	EPC	Relieves *miR-22*-induced SIRT1 downregulation by competitively sponging *miR-22*.	Ming et al., 2016
*STEEL*	Human	N/A	HUVEC; HMVEC	Binds the chromatin-associated enzyme PARP1 to assist its binding to the *KLF2* and *eNOS* promoters.	Man et al., 2018
*TGFB2-OT1*	Human	N/A	HUVEC	Binds *miR-3960, -4488, and -44459*, which target *CERS1, NAT8L*, and *LARP1*, respectively, the key proteins involved in autophagy and inflammation.	Huang et al., 2015
*tie-1AS lncRNA*	Human; mouse; Zebrafish	Zebrafish Tg(flk:EGFP)	HUVEC	Binds *tie-1* mRNA and regulates *tie-1* transcript levels, resulting in specific defects in endothelial cell contact junctions.	Li et al., 2010
*uc001pwg.1*	Human	Stenosed and nonstenotic uremic veins	HUVEC; EC derived from human-induced pluripotent stem cells	Its overexpression increases eNOS phosphorylation and NO production by affecting the expression level of nearby protein-coding gene MCAM.	Lv et al., 2017

Each lncRNA is listed with organism(s), tissue(s), cell type(s), and function(s) along with the corresponding reference. The abbreviations used are as follows: “coronary artery disease (CVD)”; “endothelial progenitor cells (EPCs)”; “human aortic endothelial cell (HAEC)”; “human aortic endothelial cells (HAoEC)”; “human brain microvascular endothelial cells (HBMVEC)”; “human dermal lymphatic endothelial cells (HDLEC)”; “human microvascular endothelial cells (HMEC)”; “human microvascular endothelial cells (HMVEC)”; “myocardial microvascular endothelial cells (mMVE)”; “human pulmonary artery endothelial cells (PAEC)”; “rat brain microvascular endothelial cells (RBMVEC)”; and “not applicable (N/A).”

In addition to the above lncRNAs, we recently reported the presence of an emerging class of lncRNAs called “circular RNAs (circRNAs)” in ECs (Boeckel et al., 2015). CircRNAs are byproducts of splicing events (more specifically, “backsplicing”) of mostly protein-coding genes (Jeck et al., 2013; Jeck and Sharpless, 2014; Boeckel et al., 2015), are stable and localized predominantly in the cytoplasm (Nigro et al., 1991; Cocquerelle et al., 1993). When some circRNAs are knocked down, there are phenotypes observed, which may not be observed when the parental transcripts of circRNAs are knocked down (Boeckel et al., 2015; Gerstner et al., 2016). For example, the hypoxia-regulated circRNA *cZNF292*, which is derived by backsplicing of *ZNF292* protein-coding gene, exhibits proangiogenic activities (Boeckel et al., 2015). Some studies suggest that circRNAs function as miRNA sponges (Hansen et al., 2013; Memczak et al., 2013; Geng et al., 2016; Liu Q. et al., 2016; Zheng et al., 2016). However, recent comprehensive bioinformatics analysis (Guo et al., 2014) and our biological validation experiments (Boeckel et al., 2015; Weirick et al., 2016b) indicate that circRNAs functioning as miRNA sponges are extremely rare. Along with lncRNAs, more studies are necessary to uncover the functions of circRNAs in ECs.

## RNA-seq data analysis using bioinformatics

There are two major methods of generating libraries for RNA-seq, which are based on poly-A selection and ribosomal RNA (rRNA)-depletion. Both methods are aimed at removing rRNAs, which constitute ~80% of total RNA followed by 15% transfer RNAs (tRNAs) and only 5% for all other RNAs, including protein-coding genes and lncRNAs (Lodish et al., 2000). The poly-A selection will result in the identification of protein-coding genes and lncRNAs with poly A tails (~60% of total lncRNAs; Cheng et al., 2005), while the rRNA-depletion can identify the rest of lncRNAs and circRNAs—in addition to those identified in the former method. The presence of circRNAs is detected only with the latter method as circRNAs arise from exons and/or introns that are spliced out, which are devoid of poly A tails.

Analysis of RNA-seq data usually involves a number of common computational steps to obtain the expression profiles of the RNA in a set of samples. At the start of a typical analysis pipeline, reads are trimmed to remove primers and low-quality regions of reads. Next, the reads are aligned to a genome in a “guided alignment.” In the case of the organism with no reference genome, a “*de novo* assembly” of the transcriptome is performed. However, *de novo* assembly is more error-prone and difficult to operate, thus we will simply focus on guided alignments. Traditionally, Tophat (Trapnell et al., 2012) has been the most popular aligner, but it is now being supplanted by newer programs (e.g., STAR, HISAT2), which offer greater speed and alignment accuracy (Engström et al., 2013; Conesa et al., 2016; Costa-Silva et al., 2017; Zhang C. et al., 2017).

Similar to protein-coding genes, lncRNAs undergo alternative splicing (AS) to produce isoforms (Deveson et al., 2017; White et al., 2017). The current understanding of AS is mainly based on EST-cDNA sequencing and short-read RNA-seq data. In the second-generation sequencing (e.g., Illumina-based short RNA-seq), long strands of cDNA must be broken into small segments to infer nucleotide sequences by amplification and synthesis (Metzker, 2010), which fall short of detecting intact full-length transcripts. To address this shortcoming, third-generation sequencing (also known as “long-read sequencing”) may be a solution. PacBio RS II (Pacific Biosciences, CA, U.S.A.) is the first commercialized third-generation sequencer, which utilizes a novel single molecule real-time (SMRT) technology (Schadt et al., 2010). Compared to second-generation sequencing, SMRT technology offers long read lengths (up to 92 kb), high consensus accuracy (free of systematic sequencing errors), and low degree of bias (even coverage across G+C content) (Nakano et al., 2017). When this technology is applied to any transcriptome (cDNA) sequencing (e.g., RNA-seq), it is called “Iso-Seq,” which can monitor AS (Abdel-Ghany et al., 2016). With Iso-Seq, the need for transcriptome assembly is eliminated as “one read = one transcript” with each transcript can be read from its 5′-end to poly A tail. Iso-Seq has been applied to various species and tissues (Singh et al., 2016; Cheng et al., 2017; Hoang et al., 2017a,b; Jiang et al., 2017; Jo et al., 2017; Kim et al., 2017; Kuo et al., 2017; Wang et al., 2017a,b, 2018; Xue et al., 2017; Zhang S. J. et al., 2017; Zulkapli et al., 2017; Filichkin et al., 2018) but not yet to ECs.

The largely unbiased manner in which RNA-seq captures information is another interesting aspect of the technology, which enables new findings via re-analysis of published data. For example, most of the RNA-seq studies have been focused on analyzing expression of protein-coding genes. As lncRNA are also present in the data sets, these data offer a rich resource for studying lncRNA expression patterns. We have developed a number of bioinformatics tools to exploit these resources (Gellert et al., 2013; Weirick et al., 2015, 2016b, 2017), including some specifically designed to identify lncRNAs and to associate their expressions in various tissues and cell types, including ECs (e.g., our database ANGIOGENES; Müller et al., 2016). Although ECs can be found throughout the human body, there are only few databases available that contain the expression profiles for genes expressed in ECs (e.g., Causal Biological Network database Boué et al., 2015, dbANGIO4 Savas, 2012, and PubAngioGen Li et al., 2015). Our ANGIOGENE is one of the few that contain the expression profiles of both protein-coding genes and lncRNAs in various ECs based on RNA-seq data. Furthermore, ANGIOGENES covers humans, mice, and zebrafish to allow for the screening of lncRNAs in the positional conserved regions (not necessary sequence-conserved) (Weirick et al., 2015).

There are many transcripts whose sequencing reads are present in RNA-seq data but are not annotated in the public databases, including NONCODE (Zhao et al., 2016), which is one of the hallmark databases for lncRNAs. Our previous study (Weirick et al., 2016a) shows that 77,656 novel isoforms of annotated reference transcripts and 102,848 intergenic transcripts are identified with 58,789 (75.70%) and 101,993 (99.17%) being predicted as non-coding, respectively, from 12 human tissues (Nielsen et al., 2014), while there are 181,434 annotated transcripts (87.13% out of 208,244 transcripts in Ensembl version 77) are expressed in at least one of 12 tissues analyzed. Although we could validate the presence of novel lncRNAs by RT-PCR experiments, many novel lncRNAs contain repetitive elements, such as microsatellites (Bidichandani et al., 1998) and short interspersed nuclear elements (SINE), including ALU elements (Häsler and Strub, 2006). Thus, it is highly recommended to consult the available methods to characterize lncRNAs (Li et al., 2014; Liu et al., 2017), including CAGE-seq to annotate the 5′-end of lncRNAs (Hon et al., 2017) and ribo-seq/ribosomal footprinting RNA-seq technology to understand the coding potential (Ruiz-Orera et al., 2014; Ji et al., 2015; Alvarez-Dominguez and Lodish, 2017) before proceeding to more functional experiments.

It is well-known that ECs are heterogeneous populations of cells as their activities and functions differ based on their physiological locations (Aird, 2012; Regan and Aird, 2012; Yuan et al., 2016). In order to understand such heterogeneity of ECs, it is important to perform single-cell RNA-seq (scRNA-seq) instead of bulk RNA-seq by using a piece of tissue or those in a culture dish. As the technique for scRNA-seq matures, the immediate problem is the data analysis, especially positioning each cell to a particular cell type in order to organize their molecular signatures matching to the anatomical location in which each cell was isolated from. For example, hearts contain multiple cell types (e.g., cardiomyocytes, ECs, fibroblasts, pericytes, and smooth muscle cells). In regards to ECs, their expression profiles may differ for those contained in the artery and vein. When such profiles are compared to ECs from other tissues (e.g., kidneys, lungs), there are some genes that are expressed at the similar level in all tissues while others are expressed specifically in ECs isolated from a particular tissue. In order to understand such hierarchical organization of cells, their corresponding cell types, and tissues, it is utmost importance that the ontology of each cell must be organized in relation to its corresponding cell type and tissue. To achieve this hierarchical and ontological organization, we recently introduced the usage of logic programing (Weirick et al., 2016b), which was applied to kidneys. Logic programming is a programming paradigm based on formal logic, using a set of logical sentences consisting of facts, rules, and queries (Eklund and Klawonn, 1992). For example, consider a transcript expressed in the renal cortex. The renal cortex is located within kidneys. When sequencing whole kidney under the same condition, the same transcript should be expressed. One could even descend to the level of cell types (e.g., ECs isolated from interlobular arteries, which are located within the kidney cortex). Similarly, all sequences expressed within these ECs are expressed in the kidney. Furthermore, it is well-known that high abundance sequences can overwhelm lower abundance sequences. Thus, logic programming can be useful for integrating RNA-seq data at different hierarchical levels and beyond. This can be accomplished by: (1) modeling the anatomical and experimental relationships; (2) creating rules to define various types of expression characteristics; and (3) using queries to determine expression characteristics of a given RNA. The analysis of RNA-seq data of ECs in the heart for lncRNAs, coupled with logic programming, should help to facilitate the further usage of the available RNA-seq data (e.g., single cell RNA-seq data from the heart) to test various hypotheses that were not originally intended when the data were generated. Such an approach should yield the identification of lncRNAs in a variety of conditions (e.g., expressed in atherosclerotic plaques but not in the healthy artery), which can be further validated in functional studies.

## Detection of RNA editing patterns from RNA-seq data

In addition to studying lncRNAs, re-analysis of publicly-available RNA-seq data is also useful for studying RNA editing. RNA editing is a post-transcriptional modification to alter the sequence of RNA molecules (Keegan et al., 2001; Hideyama and Kwak, 2011). The full extent and reasons for RNA editing is largely unknown. However, recent studies show that the editing in exons leads to an amino acid substitutions from altered codons (Alon et al., 2015; Liscovitch-Brauer et al., 2017), whereas editing in 3′-untranslated regions (UTRs) may affect binding of RNA binding proteins (RBPs) or microRNAs (miRNAs) thereby modulating RNA stability and/or translation (Keegan et al., 2001). There are two types of RNA editing: adenosine to inosine (A-to-I) and cytidine to uridine (C-to-U). A-to-I is the most common form and occurs through RNA editing enzymes called “adenosine deaminases acting on RNA (ADARs),” which convert adenosine in double-stranded RNA into inosine (Savva et al., 2012). When reverse transcribed to complementary DNA (cDNA), an inosine is converted to guanine (“G”), which can be identified by comparison to the reference genome. A number of studies have been conducted to detect RNA editing events from RNA-seq data (Bahn et al., 2012; Park et al., 2012; Peng et al., 2012; Ramaswami et al., 2012, 2013; Solomon et al., 2013), including our recent study in ECs (Stellos et al., 2016). Because of the detection from RNA-seq data, several databases for RNA editing events have been constructed to provide evidence for the frequency of RNA editing in various conditions (Kiran and Baranov, 2010; Picardi et al., 2011, 2017; Laganà et al., 2012; Ramaswami and Li, 2014; Solomon et al., 2016; Gong et al., 2017). We recently reported that cathepsin S (*CTSS*), which encodes a cysteine protease associated with angiogenesis and atherosclerosis, is highly edited (Stellos et al., 2016). Such RNA editing enables the recruitment of stabilizing RBP human antigen R (HuR) to the 3′-UTR of *CTSS* transcript, thereby controlling *CTSS* mRNA stability and expression. The RNA editing enzyme ADAR1 levels and the extent of *CTSS* RNA editing are associated with changes in CTSS levels in patients with coronary artery diseases. Our study highlights the involvement of RNA editing in cardiovascular diseases, which has not yet been investigated (Uchida and Jones, 2018). Our finding was further supported by the recent large-scale, multi-center study analyzing RNA-seq data from the NIH Common Fund's Genotype-Tissue Expression (GTEx) program, which reported that the aorta, coronary, and tibial arteries were the most highly edited tissue type among 53 body sites from 552 individuals analyzed (Tan et al., 2017).

In humans, RNA editing occurs mostly in repetitive Alu regions (Levanon et al., 2004; Peng et al., 2012), which can be found in lncRNAs as lncRNAs can also be edited (Picardi et al., 2014; Szczesniak and Makalowska, 2016; Gong et al., 2017). Although proposed but not tested extensively, the functions of lncRNAs may depend on their conformation (e.g., 3D structures), which can be affected by their primary sequences. This folding process can be influenced by a variety of factors, including (but not limited to) RNA modifications on lncRNAs, such as RNA editing. Given that RNA editing can be readily detected from RNA-seq data, more systematic analysis of RNA editing patterns is necessary, especially targeting lncRNAs in the heart (Uchida and Jones, 2018). For this purpose, several bioinformatics tools are available to detect editing within RNA-seq data, including GIREMI (Zhang and Xiao, 2015), JACUSA (Piechotta et al., 2017), RED (Sun et al., 2016), RED-ML (Xiong et al., 2017), REDItools (Picardi and Pesole, 2013), RES-Scanner (Wang et al., 2016), and our RNAEditor (John et al., 2017).

## How could we translate the concept of lncRNAs into RNA therapeutics

The one obvious usage of lncRNAs in medicine is using lncRNAs as diagnostic biomarkers as lncRNAs are more cell-type specifically expressed than protein-coding genes (Thurman et al., 2012; Gellert et al., 2013; Necsulea et al., 2014; Weirick et al., 2015). Although some progresses have been made, most of RNA-seq data analyzed so far does not consider lncRNAs due to the reasons mentioned above. Thus, without performing further RNA-seq experiments, it should be feasible to discover lncRNAs that capable of differentiating between diseased and healthy individuals by re-analyzing publicly-available RNA-seq data. For this purpose, bioinformatics tools mentioned above should be useful.

## Author contributions

All authors made contributions to survey the current status of lncRNA research. All authors approved the final version of this manuscript.

### Conflict of interest statement

The authors declare that the research was conducted in the absence of any commercial or financial relationships that could be construed as a potential conflict of interest. The reviewer KT and handling Editor declared their shared affiliation.
